# Urban (Digital) Play and Right to the City: A Critical Perspective

**DOI:** 10.3389/fpsyg.2021.636111

**Published:** 2021-03-09

**Authors:** Eunice Castro Seixas

**Affiliations:** Research Centre in Economic and Organizational Sociology (SOCIUS), Research in Social Sciences and Management (CSG), Lisbon School of Economics and Management, Universidade de Lisboa, Lisbon, Portugal

**Keywords:** urban play, right to the city, Lefebvre, playable cities, playful citizens

## Abstract

In this paper I discuss the concept of the right to the city in articulation with the concept of urban play and more specifically, the diverse body of research related with playable and playful cities. Following a brief review of these two concepts and related studies, I critically discuss the possibilities of articulating Lefebvre's radical concept of the right to the city to contemporary interventions on urban and digital play.

## Introduction

Although urban play is not a new concept, it is becoming increasingly pervasive in the language and scholarly work on place-making, design and urban planning, particularly in regard to smart or digital cities. Nevertheless, the concept of urban play encompasses a variety of meanings, perspectives and urban interventions. In this paper I discuss Lefebvre's concept of the right to the city in articulation with the concept of urban play, and more specifically, the framework of playable and playful cities.

Lefebvre (1968) concept of the right to the city has inspired numerous studies from several academic disciplines, and remains today an important concept for critical scholars with an interest in the urban question. In his work as a whole, Lefebvre offers us a complex and holistic framework for understanding urbanization and urban life in relation to the growth of capitalism. His concept of the right to the city as a right “to urban life, to renewed centrality, to places of encounter and exchange, to life rhythms and time uses, enabling the full and complete usage of these moments and places” (Lefebvre, 1996, p. 179) reveals the author's attention to the multiple aspects of human experience (Purcell, 2014). The notion of “play” is also present in Lefebvre's concept of the right to the city, as the latter “stipulates the right to meetings and gatherings (…) the need for social life and a centre, the need and the function of play, the symbolic functions of space” (Lefebvre, 1996, p. 195).

Several scholars have drawn inspiration from Lefebvre's proposals related to creative space appropriation, art and urban social movements to argue for conceiving the urban environment as a play space. These perspectives share with Lefebvre a critique of a reductionist conceptualization of play as determined by the capital—the “abstract space,” and restricted to specific designated areas in the cities. On the other hand, the radical nature of the right to the city concept as framed by Lefebvre is at odds with several of its current interpretations and applications, which tend to depoliticize his work (Busquet, 2013), and contribute to the narrative of technology as a solution to social problems (Caprotti, 2014).

## The Radical Conception of the Right to the City

The right to the city, as conceptualized by Lefebvre (1968, 1996) and Harvey (2008, 2012) is a collective right to change the city and shape the process of urbanization.

The right to the city is, therefore, far more than a right of individual access to the resources that the city embodies: it is a right to change ourselves by changing the city more after our heart's desire. It is, moreover, a collective rather than an individual right since changing the city inevitably depends upon the exercise of a collective power over the processes of urbanization (Harvey, 2008, p. 23).

Understood as a collective right, it necessarily starts from a critical awareness of urban structural inequalities, and involves social struggles for appropriating and reclaiming urban spaces. These include struggles for claiming specific rights, such as the right to housing, the right to mobility, the right to citizenship, the right to participation, the right to urban nature, or the right to rest and leisure, among other. According to Lefebvre (1996) the right to the city “manifests itself as a superior form of rights: the right to freedom, to individualization in socialization, to habitat and to inhabit” (p. 174). It is both “a cry and a demand” Lefebvre, 1968, or as Marcuse (2009) explains it, “a cry out of necessity and a demand for something more.” It is a cry by city dwellers deprived of basic human rights, but it is also a demand by those seeking to develop their own potential for creativity in an urban context (Marcuse, 2009). Thus, the notion of right to the city gains relevance in the framework of contemporary urban conflicts and political struggles, namely regarding immigration and minorities' rights (Gilbert and Dikeç, 2008), but also within citizens' demands for participation in decision-making processes (Mitchell, 2014). The latter include participating in urban planning processes and reclaiming ownership of urban and digital technology. However, Lefebvre's proposals have been appropriated in many different ways and its radical conception is absent from most contemporary initiatives allegedly inspired by it (Purcell, 2014). It thus becomes crucial to clarify what the right to the city is not (Kothari and Chaudhry, 2009), while remaining vigilant toward its multiple uses and formulations.

The right to the city cannot be equated with smart city initiatives whose main goal is that of promoting a more efficient urban management, while maintaining the status quo, or even increasing the dominance of powerful actors, under the pretense of a participatory process (Kothari and Chaudhry, 2009; Kitchin, 2015; Willis, 2019). The right to the city is also not about producing creative cities or creative consumers, initiatives grounded on the entrepreneurial discourse of neoliberal urbanism (Harvey, 1989; Peck, 2005). And neither it is about constructing uneven eco-cities projects if these are conceived as technological fixes to sustainability concerns that reproduce socioeconomic inequalities (Caprotti, 2014).

Although this radicality can be considered as the most important aspect of the conception of the right to the city, there are other aspects worth mentioning. One is the emphasis on the “lived space” or the experiences of urban inhabitants, which stresses the importance of the use value of urban space over its exchange value, and provides for a more holistic understanding of social life. Such a perspective is shared by most of the perspectives on the right to the city across the academic, policy and activist spheres (Purcell, 2014). A third aspect is its conceptualization as an open-ended, pluralist process, in line with Lefebvre's proposal of city as “an oeuvre,” a dynamic space reflecting “movement, complexity, conflicts, and contradictions” (Lefebvre, 1996, p. 53). Among these contradictions are the relationship between routine and creative play, and also between critique and creativity, which are understood by Lefebvre as productive juxtapositions, opening the possibility for new aesthetic and political senses of the urban (Gardiner, 2004; Lilliendahl Larsen and Brandt, 2018). These conceptualizations of creativity as linked, in a non-linear way, to the right to the city are often quoted but not always correctly understood from the part of contemporary authors working on (digital) urban play.

## Urban (Digital) Play, Playable Cities and the Playful Citizen

The pervasiveness of urban play is visible in the growing number of initiatives for making urban space and infrastructure more playable and citizens more playful. Play is now linked to multiple values, such as health, learning, efficiency, sociability, creativeness, experimentation, innovation and even subversion. Nonetheless, play, “urban play,” playfulness and playability remain controversial concepts, “difficult to define and hard to distinguish” (Nijholt, 2017, p. 7). Urban play has been used, for example, to describe spontaneous and non-instrumental play, not only of children, but also of young people and adults. Some examples are young people's hanging out practices or parkour (Ameel and Tani, 2012; Pyyry and Tani, 2019). These can be considered as underground practices, and examples of city appropriation and reclaiming, in line with the concept of the right to the city. They highlight the disruptive and subversive potential of urban play, which has also been linked to the playfulness of various protests and social movements (Shepard, 2012; Bruttomesso, 2018), and to the practice of urban exploration (McRae, 2008). But urban play also includes forms of “digital play,” such as the sensors and actuators embedded in urban environments, gaming technologies or pervasive games, in line with the growing importance of information and communication technologies (ICT) and the trend toward digitalization of contemporary societies. This diversity of expressions of urban play reinforces an idea of play as inherently ambiguous (Sutton-Smith, 2001), justifying an inductive approach to its conceptualization (Donoff and Bridgman, 2017).

The concepts of playable and playful cities/citizens presuppose the use of smart technology to create a “digital playground in the city,” and appear in this context as critical, political and human-centered alternatives to smart cities, or at least to the technology-driven and efficiency based approaches to smart cities (Nijholt, 2017; De Lange, 2019; Innocent, 2020). In this framework, playfulness can be conceptualized “as a characteristic of human–computer interactions” that can be incorporated in the design of smartness and in making this smartness available for city dwellers (Nijholt, 2017, p. 3). The “playable city” is a broader concept that can be generally understood as “a vision of the city that makes room for play, playfulness and games as a fundamental goal of city-making” (Rivera et al., 2020, p. 91). This body of research presupposes a centrality of both play and digital technologies to contemporary urban life and it proposes, at the same time, to rethink how technology integrates with the social fabric.

The assumption that play has an intrinsic social value is highlighted by Innocent (2020), in his critique to smart cities' emphasis on datafication: “Sometimes, all that achieved is play—a problem may not be solved or identified—which is valuable in itself as play has its own intrinsic social value” (p. 28). Nonetheless, the author suggests that this issue can be overcome if the focus is on a participatory approach enabling “conversations about the city through play” (Innocent, 2020, p. 27). Bottom-up, participatory approaches have been emphasized by several authors within the frameworks of “hackable cities,” “playful citizens,” and “critical playable cities,” who have argued that such methodologies allow for translating the right to the city into practice (Anastasiu, 2019), helping citizens to reclaim ownership of urban technology (Glas et al., 2019), and pacifying urban tensions/conflicts by channeling these into peaceful creative expressions (Hassan and Thibault, 2020). As in non-digital forms of urban play, the idea of play as subversive is also incorporated in several of these proposals, specifically in the concepts of the “hackable city” (De Lange and De Waal, 2019), and “critical playable cities” (Hassan and Thibault, 2020).

It is not my goal here to make a comprehensive review of these studies on urban digital play. Rather, I would like to suggest that, despite its diversity, this research appears to share: an assumption that play has an intrinsic social value; that playfulness should not be limited to places or activities traditionally linked to play (like playgrounds or thematic parks); a preference for participatory approaches that recognize the importance of the inhabitants' lived experiences as well as their power to introduce playful applications in the urban environment that also contribute to improve their daily lives. This is nicely summed up by Nijholt (2017): “Whether a smart city is playful depends very much on how residents experience the city and how the city stimulates “playful play.” (p. 8).

The question of whether these initiatives can be understood as correctly translating Lefebvre's radical conception of the right to the city is less straightforward. Lefebvre (1991, p. 193) considers play as “a part of every human activity,” linking it to work as well as pleasure. He sees spontaneous and creative play as citizens' action (and imagination) in space, with a potential to transform the city and disrupt capitalism norms. But not all types of play hold this potential for social change, as many of its current manifestations are officially sanctioned and controlled, designed to promote private and commercialized forms of leisure, or to increase urban entrepreneurialism.

## Discussion

The right to the city should be conceptualized from a transdisciplinary, multistakeholder and inclusive perspective, building from the dialogue between civil society members, decision-makers, scholars and practitioners from several fields. However, just following a multi-stakeholder model is not enough as participation processes have often been co-opted by powerful actors within neoliberal planning. The emphasis on a fair and inclusive participatory process is a concern shared by scholars working within the framework of playable cities with the goal of co-creating “an engaging and empowering participatory place to live” (Slingerland et al., 2020, p. 3), and of fostering “civic conversation” (Innocent, 2020, p. 27).

I argue that initiatives for promoting the right to the city should not neglect the key aspects of this radical concept, specifically: (i) its understanding as a collective right for changing the city, shaping the process of urbanization and fighting for social justice; (ii) the importance of a critical analysis of the inhabitants' everyday life experiences and informal practices of appropriation in the urban spaces, including contemporary practices related to digital interactions and play in urban contexts; and (iii) the creative, unpredictable and open-ended nature of these struggles. These aspects of the right to the city concept are not easily translatable into a framework of “city-making,” nor was that the idea of Lefebvre. In fact, his conceptualization of the city as “an oeuvre” actually goes against any attempt to transform this conception into an action framework, and Lefebvre criticized models for being “abstract but serviceable representations of a projected, planned space in which some kinds of spatial practice are condoned and others dismissed as pathological” (Moravánszky et al., 2014, p. 160). Rather, the radical concept of the right to the city could be understood as an invitation to think critically about the complexity, contradictions and social struggles inherent to the process of urbanization, including the relationship between critique and creativity, and between digital and non-digital practices of space appropriations and urban play.

The growing importance of digital technologies in our societies is undeniable, and this is especially visible in the cities. That does not mean however that technology should be conceived as either a neutral solution for social problems, or as an alternative emancipatory narrative about city-making. More studies are needed in order to grasp the meaning and scope of citizens' participation in contemporary interventions related to digital cities (Cardullo and Kitchin, 2018). A focus on the fairness of the procedures should be complemented with a focus on the outcomes of participation in these smart, digital and playful cities' experiments. In this respect, there is some evidence suggesting that the goals of digital inclusion and participation, often asserted in smart city projects are sometimes pushed into the background, relatively to the goal of smart connectivity. For example, in their study of local smart city programmes across six UK cities, Cowley and colleagues have revealed a dominance of “entrepreneurial” and “service user” modes, grounded on the value of efficiency, as well as a difficulty in engaging the general public (Cowley et al., 2018). The authors show that in general, the activities oriented toward political and civic publicness tend to be less permanent than those invoking either entrepreneurial or more passive modalities, such as service-user publicness. Ultimately, we have to ask who drives the process, and especially, who benefits from these initiatives, or “whose right to the smart city” (Willis, 2019). Following Lefebvre, the question is: Do these experiments really contribute for allowing city dwellers to shape the process of urbanization and if so, in what way? And how do these interventions enable us to consider the right to the city within a larger framework, which means tackling problems related with citizenship, housing, mobility, work, education and poverty?

The thriving digital space has also revealed new social problems related with digital inequalities, surveillance, fake news, cyber-bullying, or the creation of narcissistic subjectivities, fed by social networks. Beyond the goals of developing playable cities, the right to the city should be updated in order to respond also to these newer problems. Play remains a multifaceted and disputed notion, as do conceptualizations of the “playful citizen” and “playable cities.” These cannot be assumed *a priori* nor imposed top-down within initiatives allegedly designed to promote the right to urban play. Rather than taken as an end in itself, play, including digital play, should be approached critically in its relationship with social differences and inequalities, or, in Lefebvre's proposal, creativity should be linked to critique.

Finally, and despite the importance of creativity in Lefebvre's radical conceptualization of the right to the city, these two rights—the right to the city and the right to play—have different meanings, the former being much broader than the latter, possibly also encompassing the right not to play. The pervasiveness and over-inclusiveness of the concept of play in contemporary discourse should be subjected to a critical analysis, in the same manner as Lefebvre (1968) has engaged in a critical reflection about the right to nature, and how it has been co-opted by capital and transformed into leisure. Taking inspiration from Lefebvre's dialectical logic, it could be important to explore both the right to play and the right not to play in the city, as some of the city dwellers may actually highlight other dimensions of their experiences in urban spaces besides play, like relaxation and contemplation. Like the smart city discourse that co-produces a normative “smart city citizen,” excluding those who do not fit into this framework (Vanolo, 2014), initiatives designed to promote playable/playful cities may also risk excluding citizens that do not identify with the “playful citizen” discourse.

## Data Availability Statement

The original contributions presented in the study are included in the article/supplementary material, further inquiries can be directed to the corresponding author/s.

## Author Contributions

EC, as the sole author, was responsible for reviewing and analyzing the literature and for the conceptualization and writing-up of this manuscript.

## Conflict of Interest

The author declares that the research was conducted in the absence of any commercial or financial relationships that could be construed as a potential conflict of interest.

## References

[B1] Ameel L. Tani S. (2012). Parkour: creating loose spaces? Geogr. Ann. B Hum. Geogr. 94, 17–30. 10.1111/j.1468-0467.2012.00393.x

[B2] Anastasiu I. (2019). “Unpacking the smart city through the lens of the right to the city: A taxonomy as a way forward in participatory city-making,” in The Hackable City, eds M. De Lange and M. De Waal (Singapore: Springer), 239–260. 10.1007/978-981-13-2694-3_13

[B3] Bruttomesso E. (2018). Making sense of the square: facing the touristification of public space through playful protest in Barcelona. Tourist Stud. 18, 467–485. 10.1177/1468797618775219

[B4] Busquet G. (2013). Political space in the work of Henri Lefebvre: ideology and Utopia. Spat. Justice 5, 1–12.

[B5] Caprotti F. (2014). Eco-urbanism and the Eco-city, or, denying the right to the city? Antipode 46, 1285–1303. 10.1111/anti.12087

[B6] Cardullo P. Kitchin R. (2018). Being a ‘citizen' in the smart city: up and down the scaffold of smart citizen participation in Dublin, Ireland. GeoJournal 84, 1–13. 10.31235/osf.io/v24jn

[B7] Cowley R. Joss S. Dayot Y. (2018). The smart city and its publics: insights from across six UK cities. Urban Res. Pract. 11, 53–77. 10.1080/17535069.2017.1293150

[B8] De Lange M. (2019). “The playful city: Citizens making the smart city,” in The Playful Citizen, eds R. Glas, S. Lammes, M. Lange, J. Raessens, and I. Vries (Amsterdam: Amsterdam University Press), 349–369. 10.1515/9789048535200-021

[B9] De Lange M. De Waal M. (Eds.). (2019). The Hackable City: Digital Media and Collaborative City-Making in the Network Society. Singapore: Springer Nature. 10.1007/978-981-13-2694-3

[B10] Donoff G. Bridgman R. (2017). The playful city: constructing a typology for urban design interventions. Int. J. Play 6, 294–307. 10.1080/21594937.2017.1382995

[B11] Gardiner M. (2004). Everyday utopianism: Lefebvre and his critics. Cult. Stud. 18, 228–254. 10.1080/0950238042000203048

[B12] Gilbert L. Dikeç M. (2008). “Right to the city: politics of citizenship,” in Space, Difference, Everyday Life. Reading Henri Lefebvre, eds K. Goonewardena, S. Kipfer, R. Milgrom, and C. Schmid (New York, NY; London: Routledge), 250–263.

[B13] Glas R. Lammes S. de Lange M. Raessens J. de Vries I. (Eds.). (2019). The Playful Citizen. Civic Engagement in a Mediatized Culture. Amsterdam: Amsterdam University Press. 10.1515/9789048535200

[B14] Harvey D. (1989). From managerialism to entrepreneurialism: the transformation in urban governance in late capitalism. Geogr. Ann. B Hum. Geogr. 71, 3–17. 10.1080/04353684.1989.11879583

[B15] Harvey D. (2008). The right to the city. New Left Rev. 53, 23–40.

[B16] Harvey D. (2012). Rebel Cities: From the Right to the City to the Urban Revolution. London; New York, NY: Verso books.

[B17] Hassan L. Thibault M. (2020). “Critical playable cities,” in Making Smart Cities More Playable: Exploring Playable Cities, ed A. Nijholt (Singapore: Springer), 71–85. 10.1007/978-981-13-9765-3_4

[B18] Innocent T. (2020). “Citizens of play: revisiting the relationship between playable and smart cities,” in Making Smart Cities More Playable, ed A. Nijholt (Singapore: Springer), 25–49.

[B19] Kitchin R. (2015). Making sense of smart cities: addressing present shortcomings. Camb. J. Regions Econ. Soc. 8, 131–136. 10.1007/978-981-13-9765-3_2

[B20] Kothari M Chaudhry S. (2009). Taking the Right to the City Forward: Obstacles and Promises. Nairobi: UN-Habitat.

[B21] Lefebvre H. (1968). Le Droit à La Ville. Paris: Anthropos.

[B22] Lefebvre H. (1991). Critique of Everyday Life, Vol. II. New York, NY: Verso.

[B23] Lefebvre H. (1996). Writings on Cities. Oxford: Wiley-Blackwell.

[B24] Lilliendahl Larsen J. Brandt J. (2018). Critique, creativity and the co-optation of the urban: a case of blind fields and vague spaces in Lefebvre, Copenhagen and current perceptions of the urban. Urban Plan. 3, 52–69. 10.17645/up.v3i3.1394

[B25] Marcuse P. (2009). From critical urban theory to the right to the city. City 13, 185–197. 10.1080/13604810902982177

[B26] McRae J. D. (2008). Play city life: Henri Lefebvre, urban exploration and re-imagined possibilities for urban life (Master thesis), Queen's University, Kingston, ON, Canada.

[B27] Mitchell D. (2014). The Right to the City: Social Justice and the Fight for Public Space. New York, NY: Guilford.

[B28] Moravánszky Á. Schmid C. Stanek L. (Eds.). (2014). Urban Revolution Now: Henri Lefebvre in Social Research and Architecture. Surrey: Ashgate Publishing, Ltd.

[B29] Nijholt A. (2017). Playable Cities. Singapore: Springer. 10.1007/978-981-10-1962-3

[B30] Peck J. (2005). Struggling with the creative class. Int. J. Urban Regional Res. 29, 740–770. 10.1111/j.1468-2427.2005.00620.x

[B31] Purcell M. (2014). Possible worlds: Henri Lefebvre and the right to the city. J. Urban Affairs 36, 141–154. 10.1111/juaf.12034

[B32] Pyyry N. Tani S. (2019). More-than-human playful politics in young people's practices of dwelling with the city. Soc. Cult. Geogr. 20, 1218–1232. 10.1080/14649365.2017.1358823

[B33] Rivera M. B Ringenson T. Pargman D. (2020). “The sustainable playable city: making way for the playful citizen,” in Making Smart Cities More Playable. Gaming Media and Social Effects, ed A. Nijholt (Singapore: Springer), 87–106. 10.1007/978-981-13-9765-3_5

[B34] Shepard B. (2012). Play, Creativity, and Social Movements: If I Can't Dance, It's Not My Revolution. New York, NY: Routledge. 10.4324/9780203831489

[B35] Slingerland G. Lukosch S. Brazier F. Hengst M. D. Nevejan C. (2020). Together we can make it work! Towards a design framework for inclusive and participatory city-making of playable cities. Front. Comput. Sci. 2:600654, 1–16. 10.3389/fcomp.2020.600654

[B36] Sutton-Smith B. (2001). The Ambiguity of Play. Cambridge, MA: Harvard University Press.

[B37] Vanolo A. (2014). Smartmentality: the smart city as disciplinary strategy. Urban Stud. 51, 883–898. 10.1177/0042098013494427

[B38] Willis K. S. (2019). “Whose right to the smart city?” in The Right to the Smart City, eds P. Cardullo, C. Di Feliciantonio, and R. Kitchin (Bingley: Emerald Group Publishing), 27–42. 10.1108/978-1-78769-139-120191002

